# Treatment recommendations and predictors in Eating Disorders

**DOI:** 10.1192/j.eurpsy.2024.97

**Published:** 2024-08-27

**Authors:** F. Fernandez-Aranda

**Affiliations:** ^1^Clinical Psychology, University Hospital of Bellvitge-IDIBELL; ^2^CIBERobn, ISCIII; ^3^Clinical Sciences, University of Barcelona, Barcelona, Spain

## Abstract

**Abstract:**

Eating disorders are severe mental disorders with a high mortality rate - suicidality - and a high incidence in adolescence and early adulthood, especially in women. The course of these disorders is uncertain and treatment outcomes are limited, with successful outcomes in 50-75% of cases. For bulimia nervosa (BN) and binge eating disorder (BED), several factors, such as duration of the disorder, eating and general psychopathology, dysfunctional personality traits and cognitive impairment, have been found to be associated with treatment adherence and response. In anorexia nervosa (AN) and atypical ED (OSFED), treatment response is poorer, with higher dropout rates and longer duration and chronicity. In this presentation, we will describe recent prospective observational studies in large samples of EDs analysing clinical, personality and cognitive predictors of treatment response in eating disorders, as well as potential associated neurobiomarkers. Optimisation of health care resources and transitions, as well as early and effective personalised treatments, can change the trajectory of EDs.

**Disclosure of Interest:**

F. Fernandez-Aranda Grant / Research support from: We thank CERCA Programme/Generalitat de Catalunya for institutional support. We also want to thank the Institut d’Investigació Biomèdica de Bellvitge (IDIBELL) and ISCIII (CIBERobn is its initiative). This research was supported by grants from Instituto de Salud Carlos III (ISCIII) (FIS PI20/00132) and co-funded by FEDER funds/European Regional Development Fund (ERDF), a way to build Europe.Additional support was received from the Delegación del Gobierno para el Plan Nacional sobre Drogas (2021I031) and Ministerio de Ciencia e Innovación (grant PID2021-124887OB-I00), but also AGAUR-Generalitat de Catalunya (2021-SGR-00824), European Union’s Horizon 2020 research and innovation program under Grant agreement no. 847879 (PRIME/H2020, Prevention and Remediation of Insulin Multimorbidity in Europe) and the European Union’s Horizon Europe research and innovation program under grant agreement No 101080219 (eprObes)., Consultant of: FFA received consultancy and speakers honoraria from Novo Nordisk.

